# Molecular mechanisms and physiological functions of mitophagy

**DOI:** 10.15252/embj.2020104705

**Published:** 2021-01-13

**Authors:** Mashun Onishi, Koji Yamano, Miyuki Sato, Noriyuki Matsuda, Koji Okamoto

**Affiliations:** ^1^ Laboratory of Mitochondrial Dynamics Graduate School of Frontier Biosciences Osaka University Suita Japan; ^2^ The Ubiquitin Project Tokyo Metropolitan Institute of Medical Science Tokyo Japan; ^3^ Laboratory of Molecular Membrane Biology Institute for Molecular and Cellular Regulation Gunma University Maebashi Japan

**Keywords:** autophagy, mitochondria, phosphorylation, quality and quantity control, ubiquitin, Autophagy & Cell Death

## Abstract

Degradation of mitochondria via a selective form of autophagy, named mitophagy, is a fundamental mechanism conserved from yeast to humans that regulates mitochondrial quality and quantity control. Mitophagy is promoted via specific mitochondrial outer membrane receptors, or ubiquitin molecules conjugated to proteins on the mitochondrial surface leading to the formation of autophagosomes surrounding mitochondria. Mitophagy‐mediated elimination of mitochondria plays an important role in many processes including early embryonic development, cell differentiation, inflammation, and apoptosis. Recent advances in analyzing mitophagy *in vivo* also reveal high rates of steady‐state mitochondrial turnover in diverse cell types, highlighting the intracellular housekeeping role of mitophagy. Defects in mitophagy are associated with various pathological conditions such as neurodegeneration, heart failure, cancer, and aging, further underscoring the biological relevance. Here, we review our current molecular understanding of mitophagy, and its physiological implications, and discuss how multiple mitophagy pathways coordinately modulate mitochondrial fitness and populations.

## Graphical abstract

This review describes the conserved pathways for mitochondrial degradation via selective autophagy across species, and how multiple mitophagy pathways cooperate to modulate mitochondrial fitness and number in normal or disease physiology.

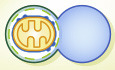

**Glossary**
ALLO‐1: Allophagy‐1
ATG: Autophagy‐related protein
BCL2L1/BCL‐XL: BCL2 like 1
BCL2L13: B‐cell lymphoma 2‐like 13
BNIP3: BCL2 and adenovirus E1B 19‐kDa‐interacting protein 3
BNIP3L: Nip3‐like protein X (NIX)/BNIP3‐like protein
CCCP: Carbonyl cyanide m‐chlorophenylhydrazone
cGAS: Cyclic GMP‐AMP synthase
CK2: Casein kinase 2
CPS‐6: Mitochondrial endonuclease G
DFCP1/ZFYVE1: DFCP1/zinc finger FYVE‐type containing 1
FIP200/RB1CC1: FIP200/RB1‐inducible coiled‐coil protein 1
Fis1: Fission, mitochondrial 1
FKBP8/FKBP38: FK506‐binding protein 8
FOXO1: Forkhead box O1
FUNDC1: FUN14 domain‐containing protein 1
GABARAP: GABA type A receptor‐associated protein
GABARAPL1/2: GABA type A receptor‐associated protein‐like 1/2
GFP: Green fluorescent protein
HOPS: Homotypic fusion and vacuole protein sorting
IGF‐1: Insulin‐like growth factor 1
Keap1: Kelch‐like ECH‐associated protein 1
LC3A/B/C: Microtubule‐associated protein 1 light chain 3 alpha/beta/gamma
LIR: LC3‐interacting region
MARCH5/MITOL: Membrane‐associated ring‐CH‐type finger 5
MBP: Maltose‐binding protein
Miro: Mitochondrial Rho
mTORC1: Mechanistic target of rapamycin complex 1
MUL1: mitochondrial E3 ubiquitin protein ligase 1
NBR1: NBR1 autophagy cargo receptor
NDP52/CALCOCO2: NDP52/calcium binding and coiled‐coil domain 2
NLRP3: NLR family pyrin domain‐containing 3
NOD: Nucleotide‐binding oligomerization domain
NRF2: Nuclear factor, erythroid 2‐like 2
OPTN: Optineurin
p62/SQSTM1: p62/Sequestosome 1
PARL: Presenilin‐associated rhomboid‐like protein
PC: Phosphatidylcholine
PE: Phosphatidylethanolamine
PGAM5: PGAM family member 5, mitochondrial serine/threonine protein phosphatase
PI: Phosphatidylinositol
PI3K: Phosphatidylinositol 3‐kinase
PI3P: Phosphatidylinositol‐3‐phosphate
PINK1: PTEN induced kinase 1
PLEKHM1: Pleckstrin homology and RUN domain‐containing M1
RABGEF1: RAB guanine nucleotide exchange factor 1
Rheb: Ras homolog, mTORC1 binding
SNARE: Soluble N‐ethylmaleimide‐sensitive factor attachment protein receptor
Src: SRC proto‐oncogene, non‐receptor tyrosine kinase
STING: Stimulator of interferon genes
TAX1BP1: Tax1 binding protein 1
TBC1D15: TBC1 domain family member 15
TBC1D17: TBC1 domain family member 17
TBK1: TANK‐binding kinase 1
TOMM/TOM: Translocase of the outer mitochondrial membrane
TORC1: Target of rapamycin complex 1
UBAN: Ubiquitin‐binding domain in ABIN proteins and NEMO
ULK1: Unc‐51‐like autophagy activating kinase 1
USP: Ubiquitin specific protease
VDAC: Voltage‐dependent anion channel
VPS: Vacuolar protein sorting
WIPI: WD repeat domain, phosphoinositide interacting

## Introduction

Mitochondria are double‐membrane‐bound subcellular compartments that function in fundamental processes such as ATP production, phospholipid biosynthesis/transport, calcium signaling, and iron homeostasis (Raffaello *et al*, 2016; Tamura & Endo, 2017; Spinelli & Haigis, 2018). These organelles act as platforms for various events including apoptosis, innate immune response, and cell differentiation (Mehta *et al*, 2017; Kalkavan & Green, 2018; Lisowski *et al*, 2018). Since mitochondria generate reactive oxygen species (ROS) from the electron transport chain, they are constantly challenged with oxidative stress that ultimately may lead to their structural and functional failure (Wong *et al*, 2017). Therefore, cells need sophisticated systems for maintaining mitochondrial fitness. Mitochondrial quality control relies on diverse pathways: ROS scavenging, DNA repair, and protein refolding/degradation (Scheibye‐Knudsen *et al*, 2015). In addition to these processes, mitochondrial fusion and fission play key roles in mitochondrial quality control (Eisner *et al*, 2018). While fusion promotes content mixing between healthy and partially dysfunctional mitochondria, fission separates damaged mitochondrial components from the mitochondrial pool.

The autophagic system targets impaired mitochondria and delivers them to lysosomes for degradation. This catabolic process, called mitophagy, contributes to maintaining mitochondrial quality control (Pickles *et al*, 2018) and mitochondrial quantity in multiple cell types. In tissues consuming a large amount of ATP such as brain, skeletal muscle, heart, liver, and kidney, mitochondria are highly developed in order to maintain the proper balance between energy demand and supply. When these cells are shifted from normoxia to hypoxia, mitophagy is induced to decrease mitochondrial quantity, thereby adapting cellular metabolism to anaerobic conditions (Wu & Chen, 2015). Thus, mitochondrial biogenesis and degradation are two opposing processes that determine mitochondrial quantity (Ploumi *et al*, 2017). In addition, mitochondria are almost completely eliminated during erythrocyte maturation (Ney, 2015). Furthermore, accumulating evidence reveals that maternal inheritance of mitochondrial DNA (mtDNA) depends on selective clearance of sperm‐derived paternal mitochondria during early embryogenesis (Sato & Sato, 2017).

**Figure 1:**
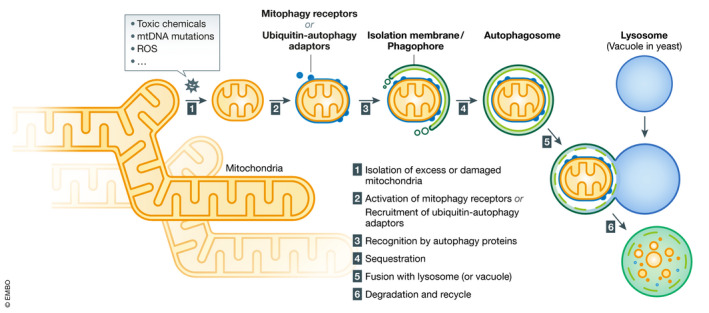
Overview of mitophagy (1) Intra‐ and extracellular cues promote isolation of excess or damaged mitochondria via fragmentation of tubular networks. (2) Mitophagy receptors or ubiquitin–autophagy adaptors that confer selectivity for degradation are recruited and/or activated on the surface of mitochondria. (3) Core autophagy‐related proteins target to mitochondria and generate the isolation membrane/phagophore surrounding mitochondria. (4) Targeted mitochondria are enclosed and sequestrated by autophagosomes. (5) Autophagosomes are transported and fused with lytic compartments such as vacuoles in yeast or lysosomes in mammals. (6) Lysosomal or vacuolar acidic hydrolases flow into autophagosomes to degrade mitochondria, and the contents will be recycled.

Although autophagy is generally recognized as a bulk degradation process that non‐selectively transports cytoplasmic components such as nucleic acids, proteins, and organelles to lysosomes (Nakatogawa, 2020), it also acts as a selective system to mediate clearance of particular organelles (Gatica *et al*, 2018). Mitophagy is one of the organelle‐specific autophagy pathways that serves to maintain cell structure and function (Okamoto, 2014) (Fig 1). The term “mitophagy” was first coined in 2005 (Lemasters, 2005; Priault *et al*, 2005), and within a few years, major breakthroughs led to the discovery of key proteins that selectively mediate mitochondrial degradation in yeast (Okamoto *et al*, 2009; Kanki *et al*, 2009b) and mammalian cells (Schweers *et al*, 2007; Narendra *et al*, 2008; Sandoval *et al*, 2008). In this review, we will describe the molecular mechanisms underlying mitophagy in yeast, worms, *Drosophila*, and mammalian cells and cover its physiological and pathophysiological functions.

## Receptor‐mediated mitophagy in yeast

### Regulation of mitophagy by Atg32

**Figure 2:**
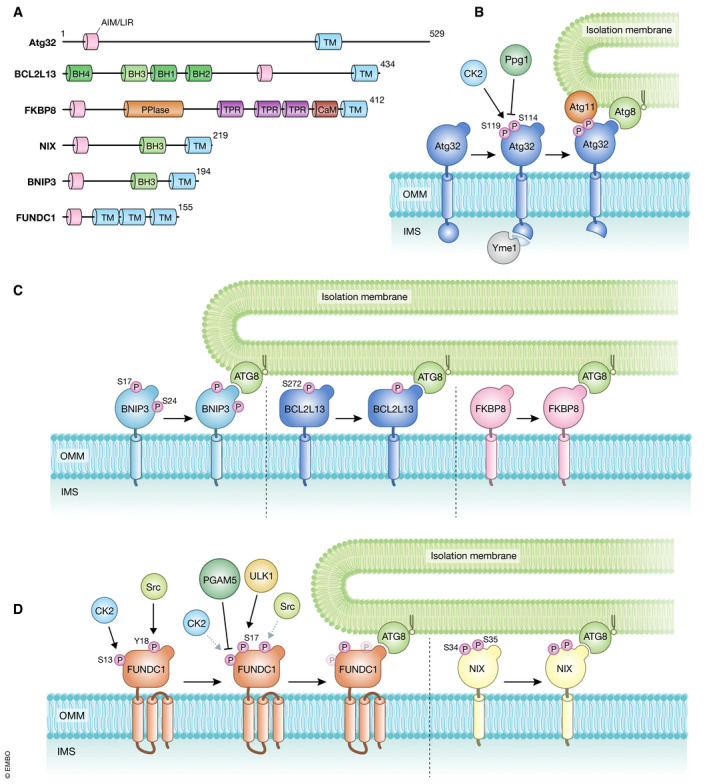
Receptor‐mediated mitophagy (A) Schematic representation of the domain structures of mitophagy receptors in yeast and mammals. AIM/LIR, Atg8‐family protein‐interacting motif/LC3‐interacting region (pink); TM, transmembrane domain (light blue); BH1‐4, Bcl‐2 homology 1‐4 domain (green and light green); PPlase, peptidyl‐prolyl cis‐trans isomerase domain (orange); TPR, tetratricopeptide repeat domain (purple); CaM, calmodulin‐binding domain (dark red). The protein size is indicated as the number of amino acids. (B‐D) Models for mitophagy receptor activation and protein recruitment on the mitochondrial surface. The yeast mitophagy receptor Atg32 (B), and the mammalian mitophagy receptors BNIP3, BCL2L13, FKBP8 (C), FUNDC1, and NIX (D) bind to ATG8 family proteins and then target the autophagy machinery to mitochondria. Phosphorylation and dephosphorylation serve as regulatory mechanisms to modulate the activity of mitophagy receptors. For details, see text.

Mitophagy in the budding yeast *Saccharomyces cerevisiae* is mostly mediated by Atg32, a single‐pass transmembrane protein in the outer mitochondrial membrane (OMM) (Okamoto *et al*, 2009; Kanki *et al*, 2009) (Fig 2A). In this unicellular eukaryote, mitophagy is induced when cells are grown to stationary phase or upon nitrogen starvation (Tal *et al*, 2007; Kanki & Klionsky, 2008; Okamoto *et al*, 2009). Under such conditions, Atg32 expression is induced at the transcriptional level and accumulates on the OMM, forming a complex with Atg8 and Atg11 on the surface of mitochondria. Atg8 is localized to autophagosomes, and Atg11 acts as a scaffold for other Atg proteins to promote autophagosome formation. Loss of Atg32 almost completely abolishes mitophagy while its overexpression increases mitophagy activity, suggesting that this molecule is a rate‐limiting factor for regulating the number of mitochondria to be degraded. Atg32 is specifically important to degrade mitochondria and is dispensable for other types of autophagy‐related processes including bulk autophagy, the cytoplasm‐to‐vacuole targeting pathway, ER‐phagy, and pexophagy.

Several lines of evidence reveal that phosphorylation is a key event for Atg32‐mediated mitophagy (Fig 2B). During respiration or upon a shift from respiration to starvation, Atg32 is phosphorylated in a manner dependent on its Atg11‐interacting motif containing Ser114 and Ser119 (Aoki *et al*, 2011; Kondo‐Okamoto *et al*, 2012). Importantly, this post‐translational modification is mediated by CK2, an evolutionarily conserved serine/threonine kinase that regulates a variety of cellular processes (Kanki *et al*, 2013). CK2 interacts with Atg32 *in vivo* and directly phosphorylates Atg32 *in vitro* (Kanki *et al*, 2013). Mutagenesis of Atg32 Ser114, Ser119, and other conserved residues in the Atg11‐interacting motif or impairment of CK2 function destabilizes Atg32‐Atg11 interactions and strongly suppresses mitophagy (Aoki *et al*, 2011; Kondo‐Okamoto *et al*, 2012; Kanki *et al*, 2013), suggesting that CK2‐dependent phosphorylation could act as a regulatory step to activate Atg32 for recruiting Atg11 to mitochondria.

A recent study has demonstrated that the protein phosphatase 2A (PP2A)‐like protein Ppg1 is critical for dephosphorylation of Atg32 and negatively regulates mitophagy (Furukawa *et al*, 2018) (Fig 2B). In cells lacking Ppg1, Atg32 is phosphorylated even at the respiratory log phase (stage prior to mitophagy induction), likely resulting in increased Atg32‐Atg11 interactions that accelerate mitochondrial degradation (Furukawa *et al*, 2018). Ppg1‐dependent mitophagy suppression also requires its binding partners Far proteins that have previously been suggested to form a complex critical for pheromone‐induced cell cycle arrest (Pracheil & Liu, 2013). These findings raise the possibility that the Ppg1‐Far complex dephosphorylates Atg32, competing against CK2‐mediated phosphorylation under mitophagy non‐inducing conditions.

Atg32 has been known to be proteolytically cleaved by Yme1, a catalytic subunit of metalloprotease in the inner mitochondrial membrane (IMM) that belongs to the ATPases associated with diverse cellular activities (AAA) protein family (Leonhard *et al*, 1996). Upon mitophagy, Atg32 is proteolytically processed at its C‐terminal portion in a Yme1‐dependent manner (Wang *et al*, 2013) (Fig 2B). Loss of Yme1 leads to a strong decrease in Atg32‐Atg11 interactions and mitophagy under nitrogen starvation (Wang *et al*, 2013). These findings support the idea that Yme1‐mediated proteolysis is required for efficient mitophagy. However, other studies suggest minor or no mitophagy deficiencies in cells lacking Yme1 (Welter *et al*, 2013; Gaspard & McMaster, 2015), raising the possibility that Yme1‐dependent processing may be relevant to Atg32‐mediated mitophagy in some specific strains and/or under some specific conditions.

### Regulation of mitophagy via ER factors

In yeast, mitochondria and the ER are connected at contact sites via the ER–mitochondria encounter structure (ERMES) complex that facilitates phospholipid transfer between these two organelles (Kornmann *et al*, 2009). The ERMES complex is localized at discrete foci where the ER and mitochondria are closely positioned, and loss of ERMES leads to severe defects in starvation‐induced mitophagy (Bockler & Westermann, 2014). Under starvation conditions, the ERMES component Mmm1 forms foci that partially co‐localize with Atg8 dot‐like structures, suggesting that autophagosomes are associated with the ER–mitochondria contact sites (Bockler & Westermann, 2014). Ubiquitylation of the ERMES component Mdm12/34 by the E3 ligase Rsp5 has also been linked to mitophagy (Belgareh‐Touze *et al*, 2017).

Atg32‐mediated mitophagy is also regulated via Get1/2 complex and Opi3, two factors associated with the ER (Sakakibara *et al*, 2015; Onishi *et al*, 2018). The Get1/2 complex is important for insertion of tail‐anchored proteins into the ER membrane (Schuldiner *et al*, 2005; Schuldiner *et al*, 2008; Wang *et al*, 2014). Loss of Get1/2 causes defects in mitophagy under respiratory conditions, while other types of autophagy‐related pathways are slightly or hardly affected (Onishi *et al*, 2018). How Get1/2 acts *in trans* to promote mitochondrial clearance remains unclear. Surprisingly, loss of Opi3, a phospholipid methyltransferase localized in the ER, leads to suppression of Atg32 induction during respiration (Sakakibara *et al*, 2015). Opi3 acts in the phospholipid biosynthesis pathway for conversion of PE into PC. Depletion of Opi3 causes aberrant elevation of glutathione levels that reduces cellular oxidative stress and thus negatively affects induction of Atg32 and mitophagy (Deffieu *et al*, 2009; Okamoto *et al*, 2009; Sakakibara *et al*, 2015). These findings raise the possibility that respiring yeast cells coordinate phospholipid methylation and mitophagy through unknown mechanisms.

## Receptor‐mediated mitophagy in mammals

In mammals, mitophagy is mechanistically more complex than in yeast and is induced by different cellular stress signals and developmental changes. Disruption of mitochondrial membrane potential is a potent trigger of mitophagy (Elmore *et al*, 2001). CCCP, a proton‐selective ionophore, and antimycin A (an inhibitor of the respiratory complex III) are commonly used to impair mitochondria and activate mitophagy. Because CCCP is highly toxic and induces non‐physiological levels of mitochondrial damage especially in neurons, antimycin A is often used to induce mitophagy in neuronal cells (Cai *et al*, 2012; Ashrafi *et al*, 2014). Both reagents trigger mitochondrial depolarization and promote accumulation of mitophagy receptors on the OMM. These receptors are integral membrane proteins that promote specific binding to mammalian Atg8 family members (LC3A/B/C, GABARAP, GABARAP‐L1/2) through a conserved LC3‐interacting regions (LIRs) and regulate the formation of isolation membranes enclosing mitochondria.

Two major types of receptors have been suggested to mediate elimination of mitochondria under physiological and pathological conditions in mammals (Fig 2A). One group includes BNIP3 and BNIP3L (also known as NIX) (Boyd *et al*, 1994; Matsushima *et al*, 1998; Chen *et al*, 1999; Vande Velde *et al*, 2000; Regula *et al*, 2002; Kubli *et al*, 2007; Schweers *et al*, 2007; Sandoval *et al*, 2008; Hanna *et al*, 2012), and the other group includes FUNDC1 (Liu *et al*, 2012). In addition, BCL2L13 is the mammalian functional counterpart of yeast receptor Atg32 (Murakawa *et al*, 2015) (Fig 2A). In the following part, we will discuss the molecular functions of mitophagy receptors in mammalian cells and the role of a family of receptors, namely FKBP proteins (Bhujabal *et al*, 2017).

### BNIP3 and NIX

BNIP3 is required for efficient turnover of mitochondria under hypoxic conditions (Zhang *et al*, 2008). In response to hypoxia, BNIP3 is upregulated and anchored to the OMM via its C‐terminal transmembrane (TM) domain, exposing the N‐terminal domain to the cytosol (Hanna *et al*, 2012). BNIP3 is usually expressed as an inactive monomer in the cytosol, but following stress signals, it forms a stable homodimer via its C‐terminal TM domain and is integrated into the OMM (Chen *et al*, 1997; Ray *et al*, 2000; Kubli *et al*, 2008). BNIP3 mutations, which disrupt homodimerization but do not affect mitochondrial localization, cause a mitophagy defect, supporting the idea that homodimerization of BNIP3 is important for efficient degradation of mitochondria (Hanna *et al*, 2012). Similar to other mitophagy receptors, BNIP3 has a LIR motif at its N‐terminal region (Fig 2A) and mutations in this region block the interaction with LC3, leading to mitophagy defects. Phosphorylation of BNIP3 at Ser17 and Ser24 near the LIR motif is important for BNIP3‐LC3 interactions (Zhu *et al*, 2013) (Fig 2C).

NIX shows homology to BNIP3 (53–56% amino acid sequence identity) (Matsushima *et al*, 1998; Chen *et al*, 1999) and promotes selective degradation of mitochondria during reticulocyte maturation (Schweers *et al*, 2007; Sandoval *et al*, 2008). During erythroid differentiation, cell nucleus, mitochondria, and other intracellular organelles are eliminated, so that erythrocytes can keep maximum space for hemoglobin that delivers oxygen (Koury *et al*, 2005; Yoshida *et al*, 2005; Fader & Colombo, 2006). With the high sequence similarity between these two proteins, expression of BNIP3 can restore mitochondrial clearance in reticulocytes lacking NIX (Zhang *et al*, 2012). NIX contains an LIR motif that promotes binding to LC3A, LC3B, GABARAP, GABARAP‐L1, and GABARAP‐L2 (Novak *et al*, 2010) (Fig 2A). In CCCP‐treated cells, NIX recruits GABARAP‐L1 to damaged mitochondria and promotes mitophagy in a manner dependent on its LIR motif (Novak *et al*, 2010). Phosphorylation of Ser34 and Ser35, two tandem serine residues near the LIR motif, stabilizes NIX‐LC3 interactions and promotes mitophagy (Rogov *et al*, 2017) (Fig 2D). Similar to BNIP3, dimerization of NIX, which is regulated by phosphorylation of its C‐terminal region, is important for efficient recruitment of the autophagic machinery to mitochondria (Marinkovic *et al*, 2020).

Accumulation of ROS (triggered by oxidative phosphorylation) promotes NIX‐mediated mitophagy via a recruitment of LC3 to mitochondria (Melser *et al*, 2013). Under conditions of oxidative phosphorylation, Rheb, a small GTPase of the Ras superfamily, translocates to mitochondria and forms a complex with NIX and LC3 to promote mitophagosome formation (Melser *et al*, 2013). Expression of Rheb in HeLa cells increases mitochondrial respiration, and loss of Rheb decreases the oxygen consumption capacity (Melser *et al*, 2013). Whether these phenotypes depend on Rheb‐induced mitophagy remains to be addressed. BNIP3 has also been shown to bind and inhibit Rheb, which is crucial for mTORC1 activation (Li *et al*, 2007). As mTORC1 negatively regulates bulk autophagy and mitophagy (Bartolome *et al*, 2017), BNIP3‐dependent mTORC1 inhibition might facilitate mitophagy induction or take part in a positive feedback loop to amplify the initiation signal of mitophagy.

Several studies have reported that BNIP3 and NIX act in PINK1/Parkin‐mediated mitophagy. NIX is ubiquitylated by Parkin, which in turn promotes targeting of the selective autophagy adaptor NBR1 that binds both ubiquitin and LC3/GABARAP to promote formation of autophagosomes surrounding mitochondria (Gao *et al*, 2015). In addition, BNIP3 interacts with PINK1 and facilitates accumulation of PINK1 on the OMM, resulting in Parkin translocation to mitochondria (Zhang *et al*, 2016a). NIX also contributes to CCCP‐induced mitochondrial depolarization, and accumulation of Parkin on damaged mitochondria (Ding *et al*, 2010b). Pathophysiological relevance of BNIP3 and NIX in Parkinson’s disease remains unknown.

#### FUNDC1

FUNDC1 is an integral OMM protein that functions as a receptor for hypoxia‐induced mitophagy. It contains a typical LIR motif near the N‐terminal region and three TM domains (Liu *et al*, 2012) (Fig 2A). Mutations in the LIR motif disrupt FUNDC1‐LC3 interactions and mitophagy induction (Liu *et al*, 2012). FUNDC1 protein levels are regulated in part by OMM‐anchored MARCH5/MITOL (Chen *et al*, 2017), an E3 ubiquitin ligase that is known to ubiquitylate several proteins acting in mitochondrial dynamics (Yonashiro *et al*, 2006; Sugiura *et al*, 2013; Park *et al*, 2014). FUNDC1 expression is decreased during hypoxia in a ubiquitin–proteasome‐dependent manner due to MARCH5‐mediated ubiquitylation of FUNDC1 at Lys119 (Chen *et al*, 2017). Knockdown of endogenous *MARCH5* or overexpression of a MARCH5 catalytic mutant impairs ubiquitylation and degradation of FUNDC1, thereby enhancing hypoxia‐induced mitophagy (Chen *et al*, 2017). Similar to Atg32 in yeast cells, FUNDC1 is regulated via phosphorylation and dephosphorylation during mitophagy on residues Ser13 and Tyr18 that are located near the LIR motif. Under normoxia conditions, Ser13 is phosphorylated by CK2, while the Src tyrosine kinase mediates phosphorylation of Tyr18 to negatively regulate FUNDC1‐LC3 interactions (Liu *et al*, 2012; Chen *et al*, 2014) (Fig 2D). Upon hypoxia, Src becomes inactivated, causing decreased phosphorylation of Tyr18, stabilization of the interaction between FUNDC1 and LC3, and promotion of mitophagosome formation (Liu *et al*, 2012). The mitochondrial serine/threonine phosphatase PGAM5 dephosphorylates Ser13 and enhances FUNDC1‐LC3 interactions to promote mitophagy (Chen *et al*, 2014).

Hypoxia or mitochondrial depolarization induces ULK1 expression and its targeting to mitochondria, leading to FUNDC1 phosphorylation at Ser17 (near the LIR motif) and stabilization of its interaction with LC3 (Wu *et al*, 2014b). Expression of a FUNDC1 variant defective in ULK1 binding inhibits targeting of ULK1 to mitochondria and mitophagy, suggesting that FUNDC1 also acts as a receptor for ULK1 (Wu *et al*, 2014b). Under normoxic conditions, BCL2L1/Bcl‐xL, an antiapoptotic BH3 domain‐containing molecule, binds PGAM5 and inhibits PGAM5‐FUNDC1 interactions to prevent dephosphorylation of FUNDC1 Ser13 and mitophagy (Wu *et al*, 2014a).

#### BCL2L13

Atg32 homologs have so far not been identified in mammalian cells, but findings from yeast reveal that BCL2L13 can induce mitophagy in cells lacking Atg32, raising the possibility that BCL2L13 acts as a mammalian Atg32 functional counterpart (Murakawa *et al*, 2015). BCL2L13 is an OMM‐anchored single‐pass membrane protein containing two LIR motifs (Fig 2A). BCL2L13 also regulates mitochondrial morphology and its overexpression induces mitochondrial fragmentation, while its silencing causes mitochondrial elongation (Murakawa *et al*, 2015). BCL2L13‐dependent mitophagy in yeast cells lacking Atg32 is likely mediated via the conventional autophagy machinery as it requires Atg7, a core protein essential for Atg8 lipidation (Murakawa *et al*, 2015). In addition, mutations in the second LIR motif reduce BCL2L13‐dependent mitochondrial degradation in the absence of Atg32, supporting the notion that BCL2L13 promotes mitophagy via Atg8 in yeast (Murakawa *et al*, 2015). BCL2L13 phosphorylation also seems to contribute to regulation of BCL2L13‐LC3 interactions as the mutation at Ser272 near the second LIR motif reduces mitophagy (Murakawa *et al*, 2015) (Fig 2C). BCL2L13 also interacts with ULK1 to localize the autophagy initiation complex to mitochondria (Murakawa *et al*, 2019). However, under which physiological conditions BCL2L13 is induced and activated remains to be elucidated.

#### FKBP8

The immunosuppressant drug FK506 (also known as tacrolimus) binds to a conserved family of proteins called FKBP that functions in different cellular processes including transcription, protein folding/trafficking, signaling, and apoptosis (Bonner & Boulianne, 2017). Co‐overexpression of FKBP8 and LC3A promotes degradation of depolarized mitochondria in CCCP‐treated, Parkin‐depleted HeLa cells (Bhujabal *et al*, 2017). FKBP8 is an integral OMM protein containing a canonical LIR motif near the N‐terminus and a TM domain at the C‐terminus (Fig 2A). FKBP8 preferentially interacts with LC3A over other Atg8 family proteins *in vivo*, and this is critical for its mitophagy activity (Bhujabal *et al*, 2017) (Fig 2C). Moreover, FKBP8 can escape from degradation‐prone mitochondria and localizes to the ER via unknown mechanisms (Saita *et al*, 2013; Bhujabal *et al*, 2017). Given the complexity due to its versatile functions (Bonner & Boulianne, 2017), further studies are needed to clarify whether endogenous FKBP8 is directly involved in mitophagy.

## Ubiquitin‐mediated mitophagy

### PINK1 and Parkin

Parkinson’s disease (PD) is a major neurodegenerative disease characterized by cell death of dopaminergic neurons (Lotharius & Brundin, 2002). PD occurs sporadically in 1–2% of people above 65 years of age but can also arise earlier mostly due to genetic mutations. Common disease phenotypes observed in PD patients are motor symptoms (tremor, bradykinesia, rigidity, and postural instability) that result from dopaminergic neuronal loss in *substantia nigra*. Non‐motor symptoms such as autonomic dysfunction, neuropsychiatric problems, and sleep difficulties are also frequently observed. The relationship between sporadic PD and mitochondrial abnormality has been suggested since 1980s (Corti *et al*, 2011). The serine–threonine kinase *PINK1* and the E3 ubiquitin ligase *PARKIN* were identified as causal genes for hereditary recessive PD with young onset (Kitada *et al*, 1998; Valente *et al*, 2004).

### Parkin activation

In 2008, a key study revealed that loss of the mitochondrial membrane potential triggers recruitment of Parkin to mitochondria and that Parkin promotes degradation of damaged mitochondria through autophagy (Narendra *et al*, 2008). PINK1 has subsequently been reported to regulate Parkin E3 activity upon mitochondrial depolarization (Matsuda *et al*, 2010; Narendra *et al,* 2010). Since conversion of Parkin from inactive to active form requires PINK1, PINK1‐mediated phosphorylation should play an important role in Parkin activation. PINK1 directly phosphorylates and activates Parkin on Ser65 in its ubiquitin‐like (Ubl) domain, and this phosphorylation is important for Parkin function (Kondapalli *et al*, 2012; Shiba‐Fukushima *et al*, 2012; Iguchi *et al*, 2013). However, phosphomimetic mutation did not cause autoubiquitylation of GFP‐tagged phosphomimetic Parkin, suggesting that Parkin phosphorylation itself is insufficient for its activation. Three groups independently found another PINK1 target that is key for Parkin activation, and discovered that (i) PINK1 phosphorylates ubiquitin at Ser65; (ii) ubiquitin‐derived Ser65 phosphopeptide can be detected in cells accumulating PINK1 on depolarized mitochondria; and (iii) the phospho‐ubiquitin accelerates Parkin E3 ligase activity *in vitro* (Kane *et al*, 2014; Kazlauskaite *et al*, 2014; Koyano *et al*, 2014). Recent advances in structural information and molecular mechanisms underlying Parkin activation are described in detail in Box 1.

Box 1 **Molecular mechanisms of Parkin activation on depolarized mitochondria**

Parkin consists of Ubl (ubiquitin‐like), RING0 (really interesting new gene 0), RING1, IBR (in‐between‐RING), REP (repressor element of Parkin), and RING2 domains (A). Structural analysis of Parkin alone (equivalent to a latent E3 form at steady‐state conditions) revealed that Parkin has an auto‐inhibited conformation mediated by multiple domain–domain interactions. Namely, the RING0 (also referred to as UPD; unique parkin domain) occludes the catalytic core residue Cys431 (ubiquitin acceptor site) in the RING2, and the REP binds the RING1 to block its E2‐binding interface (B‐1) (Riley *et al*, 2013; Trempe *et al*, 2013; Wauer & Komander, 2013; Kumar *et al*, 2015). Interestingly, when phosphorylated ubiquitin interacts with Parkin (B‐2), intramolecular structural remodeling takes place. Helix of the RING1 (H3) is straightened by phosphorylated ubiquitin, which induces conformational changes in RING1 and IBR, thereby releasing the Ubl (B‐2) (Kazlauskaite *et al*, 2015; Kumar *et al*, 2015; Sauve *et al*, 2015; Wauer *et al*, 2015a; Yamano *et al*, 2015). Consequently, the Ubl becomes more mobile and is phosphorylated more easily by PINK1 at Ser65 (B‐3). The phosphorylated Ubl localizes proximal to RING0/UPD as phosphorylated Ser65 of Ubl interacts with a positively charged pocket made by Lys161, Arg163, and Lys211 in RING0/UPD (B‐4) (Gladkova *et al*, 2018; Sauve *et al*, 2018). The RING2 is then flipped out and liberated from suppression by the RING0/UPD, and the catalytic center Cys431 (which is hitherto hidden in the molecule) becomes exposed (B‐4). Simultaneously, the E2 interaction surface in the RING1 (which is usually concealed by the REP) is also uncovered (B‐4). Ubiquitin‐carrying E2 binds the RING1 (B‐5), and the RING2 receives ubiquitin via a thioester linkage from E2, and finally, ubiquitin is transferred to a substrate (B‐6). By such cascading structural remodeling, Parkin is converted from a self‐inhibited dormant enzyme to an active E3.

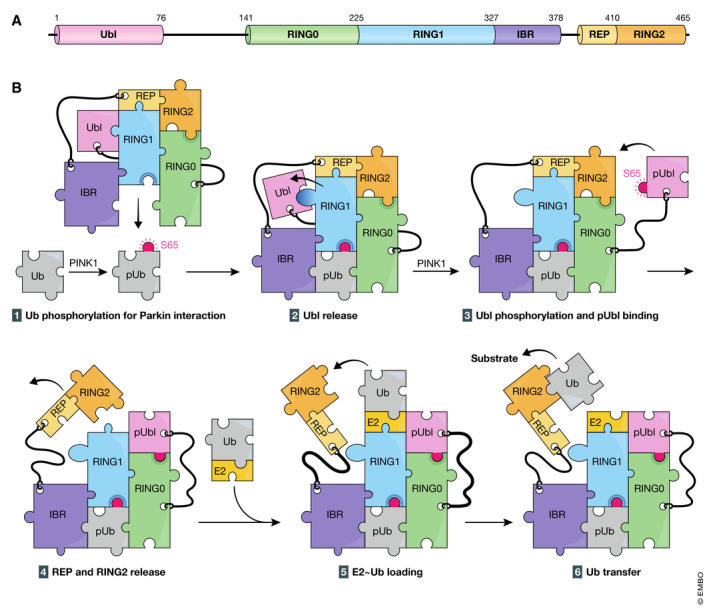

### Autophosphorylation of PINK1 is essential for ubiquitin recognition

**Figure 3:**
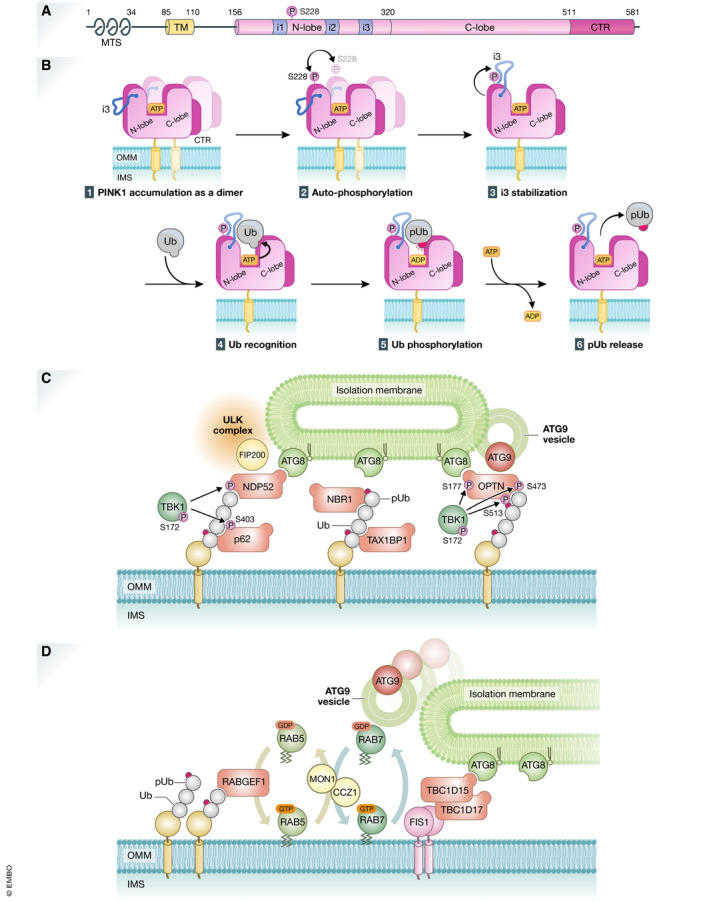
PINK1 and ubiquitin‐mediated mitophagy (A) Schematic depiction of the domain structures of PINK1. The protein and domain sizes are indicated as the number of amino acids. MTS, mitochondrial targeting signal; TM, transmembrane segment; N‐lobe and C‐lobe, N‐terminal and C‐terminal lobes found in a typical kinase, respectively. i1, i2, and i3, the insert regions unique to PINK1; CTR, C‐terminal region conserved among PINK1 homologs. (B) Molecular mechanisms underlying ubiquitin phosphorylation by PINK1 on depolarized mitochondria. (1) PINK1 forms a dimer on damaged mitochondria. (2) Ser228 is phosphorylated via intermolecular autophosphorylation in dimerized PINK1. (3) Ser228 phosphorylation stabilizes and underpins “insert 3 (i3)” at the correct position. (4) Ubiquitin (Ub) is recognized by PINK1 as a genuine substrate, (5) Ub Ser65 residue is phosphorylated via ATP hydrolysis, and (6) phosphorylated Ub (pUb) is released. (C) Recruitment of the core autophagy proteins and isolation membranes to mitochondria during PINK1/Parkin‐mediated mitophagy. Poly‐ubiquitin chains on damaged mitochondria are recognized directly by various autophagy adaptors. They are phosphorylated by TBK1 kinase, and the phosphorylation enhances the binding affinity to ubiquitin chains and ATG8 family proteins. NDP52 and OPTN specifically recruit ULK complex via FIP200 and ATG9 vesicles, respectively. (D) RABGEF1 recruited to mitochondria by poly‐ubiquitin chains triggers endosomal Rab cycles including RAB5 and the MON1/CCZ1 complex. MON1/CCZ1 directs RAB7 to mitochondria, and RAB7 facilitates the assembly of ATG9 vesicles to the autophagosome formation site. Mitochondrial Rab‐GAPs, TBC1D15 and TBC1D17, assist to complete RAB7 cycles and interact with ATG8 family proteins to recruit the isolation membrane.

PINK1 Ser228 and Ser402 residues are autophosphorylated upon decreased mitochondrial membrane potential, and this autophosphorylation is essential for Parkin recruitment onto damaged mitochondria (Okatsu *et al*, 2012). The significance of autophosphorylation at Ser402 is still unknown, and this phosphorylation site does not exist in insect PINK1. Autophosphorylation of Ser228 has been shown in both mammalian cells and insects (Woodroof *et al*, 2011). In the kinase domain, PINK1 has three unique insert regions called Insert 1, Insert 2, and Insert 3 (Fig 3A). Insert 1 varies in length from 35 to only 5 amino acids in human and in insect PINK1, respectively, and Insert 2 is not well‐conserved. By contrast, Insert 3 is highly conserved from insects to humans. The structures of the kinase and C‐terminal region (CTR) domains of insect PINKs—TcPINK (small beetle *Tribolium castaneum*) and PhPINK1 (*Pediculus humanus corporis*)—have been solved (Kumar *et al*, 2017; Schubert *et al*, 2017; Okatsu *et al*, 2018). The kinase domain consists of an N‐lobe containing five β‐sheets and a C‐lobe containing α‐helices that are connected by a hinge region. The ATP binding site and enzymatic catalytic center localize in groove between N‐lobe and C‐lobe. These features are basically common to other kinases. As a characteristic structure of PINK1, the CTR domain consists of four α‐helices that support the C‐lobe structure from backside. The structural analysis of the PhPINK1–ubiquitin complex revealed that Insert 3 is a key motif for PINK1 to recognize ubiquitin (Schubert *et al*, 2017). Phosphorylated PhPINK1 Ser202 (corresponding to human HsPINK1 Ser228) interacts with Insert 3 Arg282/Asn283 to proper position Insert 3 for ubiquitin recognition and subsequent phosphorylation (Fig 3B). As Ser202 locates on the upper side of N‐lobe and far from the enzymatic active center of PhPINK1, this seems not to involve intramolecular autophosphorylation but rather autophosphorylated *in trans* via intermolecular phosphorylation. Indeed, dimerization of HsPINK1 on depolarized mitochondria is thought to be important for autophosphorylation (Okatsu *et al*, 2013; Rasool *et al*, 2018).

### Parkin’s substrates and Ubiquitin chain amplification

PINK1‐mediated phosphorylation leads to Parkin activation and ubiquitination of substrates on damaged mitochondria that function as autophagy‐mediated degradation signals (Pickrell & Youle, 2015; Khaminets *et al*, 2016; Yamano *et al*, 2016). Upon mitophagy, several OMM proteins such as mitofusin, Miro, and VDAC have been identified as Parkin substrates (Gegg *et al*, 2010; Poole *et al*, 2010; Tanaka *et al*, 2010; Ziviani *et al*, 2010; Geisler *et al*, 2010a; Rakovic *et al*, 2011; Wang *et al*, 2011). Other OMM proteins that undergo Parkin‐mediated ubiquitylation have later been identified by mass spectrometry (Chan *et al*, 2011; Sarraf *et al*, 2013), suggesting that Parkin can ubiquitylate a large number of proteins on the surface of mitochondria. Although general E3 ligases have stringent substrate selectivity that prevents cross‐reaction among other E3s to ensure correct substrate ubiquitylation, Parkin seems to have rather low substrate selectivity. Instead, Parkin has evolved to have spatial selectivity for depolarized mitochondria rather than substrate selectivity. Artificial mitochondria‐targeted exogenous proteins such as GFP and MBP can be ubiquitylated by Parkin (Koyano *et al*, 2019). Such a unique specificity seems optimal for Parkin to achieve efficient and quick ubiquitylation of dysfunctional mitochondria. Even under steady‐state conditions, a small amount of ubiquitin is attached to proteins on the surface of mitochondria. When PINK1 phosphorylates such ubiquitin, the resultant phospho‐ubiquitin recruits Parkin from the cytosol and activates it on depolarized mitochondria to generate more ubiquitin chains. This Parkin‐catalyzed ubiquitylation then further drives PINK1‐catalyzed ubiquitin phosphorylation, leading to formation of a positive feedback loop for PINK1‐ and Parkin‐catalyzed ubiquitylation (Ordureau *et al*, 2014; Okatsu *et al*, 2015). Low substrate specificity of Parkin might facilitate this positive feedback cycle as only a small amount of PINK1 on the OMM is needed to recruit quite a few amount of Parkin to dysfunctional mitochondria (Matsuda, 2016; Matsuda & Yamano, 2020).

### Autophagosome formation in PINK1/Parkin‐mediated mitophagy

In order to detach damaged mitochondria from a healthy network and to eliminate them, proper and selective encapsulation of damaged mitochondria by autophagosomes is required. In addition, autophagosomes containing damaged mitochondria must rapidly fuse with lysosomes to facilitate their degradation. To complete these processes, many molecules involved in autophagosome/autolysosome formation work cooperatively with PINK1 and Parkin. In starvation‐induced autophagy, formation of phagophore begins at a particular region of the ER (Hayashi‐Nishino *et al*, 2009), or at the contact sites between the ER and mitochondria (Hamasaki *et al*, 2013). Several autophagy‐related proteins are recruited to the autophagosome formation site in a hierarchical order (Itakura & Mizushima, 2010).

### Autophagy adaptors in PINK1/Parkin‐mediated mitophagy

In selective autophagy‐related processes including mitophagy, a series of autophagy adaptors (p62/SQSTM1, NBR1, NDP52/CALCOCO2, TAX1BP1, and OPTN) play important roles in selective uptake of cargoes (Johansen & Lamark, 2011; Mizushima & Komatsu, 2011; Stolz *et al*, 2014; Zaffagnini & Martens, 2016). These autophagy adaptors contain both a ubiquitin‐binding domain that recognizes ubiquitin chains conjugated to the cargoes and an LC3‐interacting region that acts to recruit phagophore membranes coated with LC3. During mitophagy, all known autophagy adaptors are recruited to damaged mitochondria in a Parkin/PINK1‐dependent manner (Lazarou *et al*, 2015). Compared to the autophagic events under starvation, different cascading reactions occur during PINK1/Parkin‐mediated mitophagy. Upon mitochondrial membrane potential dissipation, the ULK1 complex and ATG9 vesicles are recruited near damaged mitochondria even in the absence of membrane‐bound LC3 (Itakura *et al*, 2012). Loss of autophagy adaptors impairs not only recruitment of the LC3‐labeled membrane to damaged mitochondria, but also recruitment of upstream autophagy‐related proteins such as ULK1 and WIPI1 during PINK1/Parkin‐mediated mitophagy. Among five autophagy adaptors, only NDP52 and OPTN can grow isolation membrane through an ATG8‐dependent positive feedback loop (Padman *et al*, 2019). In addition, NDP52 directly binds to FIP200/RB1CC1, a ULK1 complex subunit (Vargas *et al*, 2019), and OPTN can form a complex with ATG9 vesicles (Yamano *et al*, 2020). Therefore, NDP52 and OPTN bind to multiple core autophagy proteins. As compared to the hierarchy of autophagy under starvation conditions (mTORC1→ULK1→LC3), Parkin‐mediated mitophagy uses the following cascading reaction: ubiquitylation→NDP52→ULK1/LC3, and ubiquitylation→OPTN→ATG9/LC3.

### TBK1 kinase in PINK1/Parkin‐mediated mitophagy

During PINK1/Parkin‐mediated mitophagy, TBK1 directly or indirectly mediates phosphorylation of all known autophagy receptors (Richter *et al*, 2016). TBK1 activity is required for efficient recruitment of OPTN and NDP52 to the ubiquitinated mitochondria (Heo *et al*, 2015) where TBK1 phosphorylates OPTN at Ser177 to increase LC3 binding affinity (Wild *et al*, 2011) and at Ser473 and Ser513 to further increase the binding of OPTN to ubiquitin chains (Heo *et al*, 2015) (Fig 3C). Thus, in addition to Parkin–PINK1–ubiquitin‐positive feedback loop, another feedback loop (ubiquitin–OPTN–TBK1) constitutes more landing sites for autophagy adaptors on damaged mitochondria. In addition, TBK1 during mitophagy blocks mitosis due to the sequestration of TBK1 from its physiological role at centrosomes (Sarraf *et al*, 2019).

### Elongation of phagophore membranes during mitophagy

Unlike starvation‐induced autophagy by which cytoplasmic components are randomly encapsulated, mitophagy requires elongation of the phagophore membrane specifically surrounding damaged mitochondria. The LIR‐containing proteins TBC1D15 and TBC1D17 are important for expansion of the phagophore membrane during mitophagy (Yamano *et al*, 2014). TBC1D15 and TBC1D17 function as GTPase‐activating proteins (GAPs) for Rab‐type GTPases regulating membrane fusion processes in vesicular trafficking (Barr & Lambright, 2010). TBC1D15 and TBC1D17 target the OMM via their receptor Fis1 (Onoue *et al*, 2013). Abnormal LC3‐labeled tubular phagophore structures are formed upon loss of TBC1D15 or Fis1 during mitophagy, but not during starvation‐induced autophagy, in mammalian cultured cells (Yamano *et al*, 2014). In addition, loss of Fis1 in *Caenorhabditis elegans* causes a PINK1‐dependent accumulation of LC3 aggregates (Shen *et al*, 2014). Both Fis1 and TBC1D15 are required for efficient OXPHOS‐induced mitophagy and for elimination of paternal mitochondria in fertilized eggs (Rojansky *et al*, 2016). Fis1‐TBC1D15/17‐Rab may be additionally required for proper formation of autophagosomes during mitophagy. RABGEF1, an upstream factor of the endosomal Rab GTPase cascade, is recruited to damaged mitochondria via ubiquitin binding downstream of Parkin. RABGEF1 directs the Rab proteins RAB5 and RAB7 to damaged mitochondria. Furthermore, depletion of RAB7 or loss of TBK1‐mediated RAB7 phosphorylation inhibits ATG9 vesicle assembly and subsequent encapsulation of mitochondria by autophagic membranes (Yamano *et al*, 2014; Heo *et al*, 2018). These results suggest that the endosomal Rab cycle on damaged mitochondria acts as a crucial regulator of mitophagy via assembling ATG9 vesicles (Fig 3D). Furthermore, other Rab‐GAPs such as TBC1D5 target ATG9A vesicles around damaged mitochondria by regulating Rab7 activity during mitophagy (Jimenez‐Orgaz *et al*, 2018).

### Autophagosome closure and autophagosome–lysosome fusion

The final step to eliminate damaged mitochondria requires fusion of autophagosomes with lysosomes. Although it has been thought that Atg8 family proteins and their conjugation systems are required for autophagosome formation, autophagosome‐like structures are formed in the absence of lipidated Atg8 family proteins (Tsuboyama *et al*, 2016). In mammals, Atg8 family consists of six different proteins divided into the LC3 (LC3A, LC3B, and LC3C) and GABARAP (GABARAP, GABARAP‐L1, and GABARAP‐L2) subfamilies. All six proteins are covalently linked to the PE via two ubiquitin‐like conjugation systems. PE‐conjugated Atg8 family proteins associate with both elongating isolation membranes and mature autophagosomes, and LC3B is widely used as an autophagic membrane marker (Kabeya *et al*, 2000; Kabeya *et al*, 2004). Atg8 family proteins are not essential for encapsulation of damaged mitochondria by autophagosomes, but required for autophagosome–lysosome fusion (Nguyen *et al*, 2016) or efficient degradation of the inner autophagosomal membrane in lysosomes (Tsuboyama *et al*, 2016). Although damaged mitochondria are properly sequestered by autophagosomal membranes in cells lacking all Atg8 family proteins, the size of autophagosomes is much smaller than that in wild‐type cells (Nakatogawa *et al*, 2007; Weidberg *et al*, 2010).

Unlike starvation‐induced autophagy, PINK1/Parkin‐mediated mitophagy may need PLEKHM1 rather than STX17, an autophagosome‐specific SNARE, for autophagosome–lysosome fusion (McEwan *et al*, 2015). PLEKHM1 contains multiple functional domains that directly bind Rab7, the HOPS complex, and Atg8 family proteins, and is required for selective and nonselective autophagy (McEwan *et al*, 2015). GABARAP subfamily proteins localize on mature autophagosome and associate with PLEKHM1 at the lysosome to facilitate autophagosome–lysosome fusion during PINK1/Parkin‐mediated mitophagy (Nguyen *et al*, 2016).

### Deubiquitylating enzymes in PINK1/Parkin‐mediated mitophagy

Ubiquitylation is a reversible process as deubiquitylating enzymes can remove ubiquitin from ubiquitylated substrates. USP8, USP15, and USP30 regulate PINK1/Parkin‐mediated mitophagy positively and negatively (Bingol *et al*, 2014; Cornelissen *et al*, 2014; Durcan *et al*, 2014; Cunningham *et al*, 2015; Liang *et al*, 2015). USP15 and USP30 deubiquitylate mitochondrial substrates to counteract Parkin‐mediated ubiquitylation and subsequent mitophagy (Bingol *et al*, 2014; Cornelissen *et al*, 2014; Cunningham *et al*, 2015; Liang *et al*, 2015). In contrast, USP8 detaches ubiquitin from autoubiquitylated Parkin, acting as a positive regulator that promotes Parkin mitochondrial targeting and accelerates mitophagy (Durcan *et al*, 2014). Although USP8 can digest ubiquitin chains of any linkage *i*n *vitro* (Faesen *et al*, 2011), it selectively removes K6‐linked ubiquitin chains from Parkin in mammalian cultured cells (Durcan *et al*, 2014). USP30 is thought to specifically digest K6‐linked ubiquitin chains through unique ubiquitin recognition mechanisms (Gersch *et al*, 2017; Sato *et al*, 2017). It remains unclear how K6‐linked ubiquitin chains of Parkin and OMM proteins are removed selectively by USP8 and USP30, respectively. Although USP15 has been suggested to trim K48‐ and K63‐linked ubiquitin chains on depolarized mitochondria (Cornelissen *et al*, 2014), the effect of USP15 on K6‐linked ubiquitin chains has not been examined. Moreover, PINK1‐mediated ubiquitin phosphorylation impedes the enzyme activities of USP8, USP15, and USP30 (Wauer *et al*, 2015b), adding a new layer of complexity to the deubiquitylation reactions. Although early studies suggested USP30 counteracts Parkin‐mediated ubiquitylation as described, recent two papers showed that ubiquitylation of the vast majority of Parkin targets is rather unaffected in *USP30* knockout cells (Ordureau *et al*, 2020; Phu *et al*, 2020). Instead, elevated ubiquitylation is observed in components of the mitochondrial translocator and intramitochondrial substrates in *USP30* knockout cells. It is possible that USP30 removes ubiquitin from import substrates and components of the mitochondrial translocator, and these processes are required for efficient translocation through the import channels. Future studies on the actions of PINK1, Parkin, and USP8/15/30 will shed light on the functions of deubiquitylating enzymes and the significance of K6‐linked ubiquitylation in PINK1/Parkin‐mediated mitophagy.

### Parkin alternatives in mitophagy

Many papers reported that PINK1/Parkin‐catalyzed ubiquitylation induces mitophagy of damaged mitochondria. However, most of these data were obtained from experiments using cultured cells (*e.g.,* Parkin‐expressing HeLa cells), and there is much less evidence for PINK1/Parkin‐mediated mitophagy *in vivo*. In the case of genetic studies using *Drosophila*, it is controversial whether PINK1/Parkin‐catalyzed ubiquitylation induces mitophagy or not. One study reported that age‐dependent rise in mitophagy activity is abrogated in PINK1‐ or Parkin‐deficient flies (Cornelissen *et al*, 2018), whereas another work showed that any substantial impact on basal mitophagy was not observed in pink1 or parkin‐null flies (Lee *et al*, 2018). Transgenic mice to monitor mitophagy have already been established, and loss of PINK1 did not influence basal mitophagy activities in such mice (McWilliams *et al*, 2018). This finding seemingly suggests that PINK1 and Parkin are not involved in mitophagy *in vivo;* however, the results can be interpreted in several ways. Unlike human, whose dysfunction of PINK1 or Parkin causes early‐onset Parkinsonism, disease‐relevant phenotypes have not been observed in pink1 or parkin knockout mice. It might not be surprising even if mitophagy activity is normal in pink1 KO mice without an obvious phenotype. To reconcile these conflicting findings, we have to consider functional redundancy of other mitochondrial E3 ligases. Indeed, ARIH1/HHARI (Villa *et al*, 2017), March5 (Chen *et al*, 2017), MAPL/MULAN/GIDE/MUL1 (Ambivero *et al*, 2014; Yun *et al*, 2014; Li *et al*, 2015; Igarashi *et al*, 2020), p62‐keap1‐Rbx1 axis (Yamada *et al*, 2018), and HUWE1 (Di Rita *et al*, 2018) have been reported to mediate Parkin‐independent mitophagy. These E3s could compensate for PINK1/Parkin‐mediated mitophagy and conceal the output when the PINK1/Parkin function is inhibited.

## Mitophagy in worms and flies

### PINK‐1/PDR‐1‐mediated mitophagy in somatic cells

**Figure 4:**
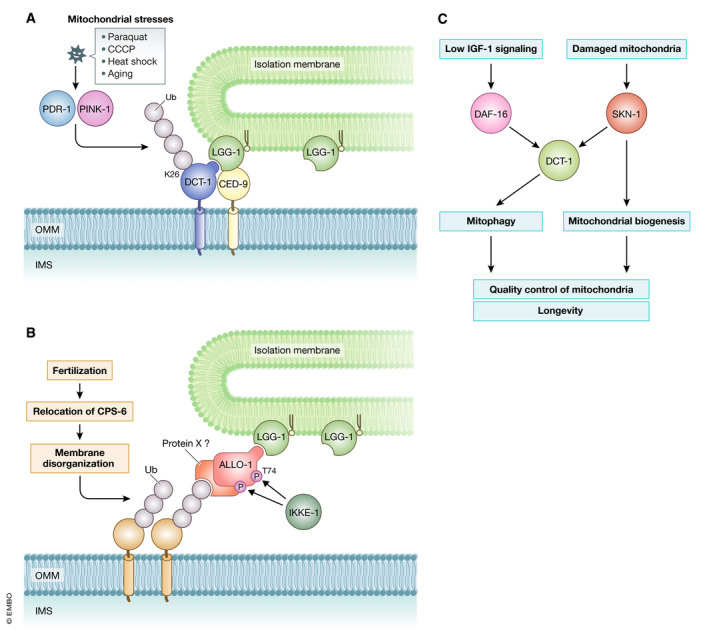
Regulation of mitophagy in *C. elegans* (A) PINK‐1/PDR‐1‐mediated mitophagy in somatic cells. DCT‐1 functions as an autophagy adaptor in association with CED‐9. DCT‐1 Lys26 (K26) is ubiquitinated in a PINK‐1/PDR‐1‐dependent manner. (B) Mechanism of allophagy in embryos. Fertilization triggers relocation of CPS‐6, the mitochondrial endonuclease, leading to membrane disorganization and ubiquitylation of paternal mitochondria. Ubiquitin (Ub) molecules on paternal mitochondria are recognized directly or indirectly by the autophagy adaptor ALLO‐1. IKKE‐1‐dependent phosphorylation of ALLO‐1 is also important for allophagy. (C) Transcriptional regulation of DCT‐1 contributes to mitochondrial homeostasis and longevity.

In the nematode *Caenorhabditis elegans*, stress‐induced mitophagy is regulated by PINK‐1 and PDR‐1 (a worm Parkin homolog), supporting that the PINK1/Parkin‐dependent pathway has been conserved during evolution (Palikaras *et al*, 2015) (Fig 4A). The NIX and BNIP3 homolog DCT‐1 functions as an autophagy receptor for PINK‐1/PDR‐1‐mediated mitophagy (Palikaras *et al*, 2015). DCT‐1 is ubiquitylated on its Lys26 residue, and this modification is enhanced under mitophagy‐inducing conditions in a PINK‐1‐dependent manner (Palikaras *et al*, 2015). In addition to DCT‐1, the Bcl‐2 homolog CED‐9 interacts with DCT‐1 and may act in the same genetic pathway to control mitophagy (Palikaras *et al*, 2015).

### Clearance of paternal mitochondria

Besides mitophagy in somatic cells, paternal mitochondria provided by sperm are selectively degraded via autophagy in *C. elegans* fertilized embryos (Al Rawi *et al*, 2011; Sato & Sato, 2011). This type of mitophagy is a developmentally programmed process and does not require any artificial stimuli to be induced and is referred to as allogeneic (non‐self) organelle autophagy (allophagy) (Al Rawi *et al*, 2012; Sato & Sato, 2012) since the paternal organelle, so‐called membranous organelles (MOs), are also degraded in this process (Al Rawi *et al*, 2011; Sato & Sato, 2011). mtDNA is maternally inherited in many organisms including humans (Ankel‐Simons & Cummins, 1996; Sato & Sato, 2013; Sato & Sato, 2017). In worm mutants of core Atg genes, paternal mitochondria and their mtDNA persist in late‐stage embryos or even in F1 larvae, suggesting that allophagy is required to prevent transmission of paternal mtDNA to the progeny (Al Rawi *et al*, 2011; Sato & Sato, 2011). Autophagy‐dependent degradation of paternal mitochondria also occurs in *Drosophila* and mouse embryos (Politi *et al*, 2014; Rojansky *et al*, 2016).

*Caenorhabditis elegans* has two Atg8 family members, LGG‐1 and LGG‐2 that are both recruited to allophagosomes (autophagosomes containing paternal mitochondria and/or MOs). In *lgg‐2* mutant embryos, LGG‐1‐positive allophagosomes are formed, but their turnover is delayed (Manil‐Segalen *et al*, 2014; Djeddi *et al*, 2015). LGG‐2 directly binds to VPS‐39, a subunit of the HOPS complex, and enhances fusion of autophagosomes with lysosomes (Manil‐Segalen *et al*, 2014). LGG‐2 is also required for microtubule‐dependent migration of autophagosomes toward the pericentrosomal region where lysosomes are concentrated (Djeddi *et al*, 2015). In addition to autophagy‐related genes, degradation of paternal mtDNA is delayed by knockdown of proteasome subunit genes, suggesting that the ubiquitin–proteasome system is involved in this process (Zhou *et al*, 2011).

Electron tomography has revealed that the inner membrane structure of paternal mitochondria starts to be disorganized quickly after fertilization (Zhou *et al*, 2016). The OMM rapture and reduced membrane potential were also observed (Zhou *et al*, 2016). These changes in paternal mitochondrial structure are initiated before autophagosome formation and lysosomal degradation. Such qualitative alteration of paternal mitochondria could be a trigger to promote their selective autophagic clearance. CPS‐6, a mitochondrial endonuclease G, is also linked to clearance of paternal mitochondria (Zhou *et al*, 2016). CPS‐6 was originally identified as an apoptotic factor that redistributes from mitochondria to the nucleus and mediates chromosome fragmentation during apoptosis (Parrish *et al*, 2001). When paternal *cps‐6* is mutated, clearance of paternal mitochondria is delayed (Zhou *et al*, 2016). Since CPS‐6 in paternal mitochondria relocates from the mitochondrial intermembrane space to the matrix after fertilization, mtDNA digestion by matrix‐localized CPS‐6 might initiate degeneration of mitochondrial membranes (Zhou *et al*, 2016). The IMM protein prohibitin 2 also plays a role in the selective engulfment of paternal mitochondria by autophagosomes and functions as an autophagy receptor for damaged mitochondria in mammals and paternal mitochondria in *C. elegans* (Wei *et al*, 2017).

More recently, a novel LIR‐containing protein named ALLO‐1 has been identified as an autophagy adaptor for degradation of paternal mitochondria and MOs (Sato *et al*, 2018) (Fig 4B). In *allo‐1* mutant embryos, allophagosomes are not formed, and paternal organelles and paternal mtDNA remain in the late embryos or larvae (Sato *et al*, 2018). ALLO‐1 is conserved only in nematode species; however, its function is very similar to that of known autophagy adaptors. Several lines of evidence suggest that ubiquitylation of targets is involved in ALLO‐1 localization (Al Rawi *et al*, 2011; Sato & Sato, 2011). It is also reported that simultaneous knockdown of *ubc‐16* and *ubc‐18* impairs allophagy (Molina *et al*, 2019). Since mutations in the *pink‐1* or *pdr‐1* gene do not significantly affect allophagy, it remains unknown how this ubiquitylation is regulated (Sato *et al*, 2018). In addition to ALLO‐1, the worm homolog of mammalian TBK1/IKKε kinases IKKE‐1 is essential for allophagy (Sato *et al*, 2018) (Fig 4B). IKKE‐1 phosphorylates ALLO‐1 on Thr74 although additional phosphorylation targets are likely to exist (Sato *et al*, 2018). This is reminiscent of TBK1 function in mitophagy and xenophagy, and phosphorylation of adaptor molecules could be a conserved mechanism regulating selective autophagy pathways (Fig 4B).

In *Drosophila*, paternal mitochondria form a very long shape parallel to the axoneme that is degraded by multiple‐step mechanisms (Politi *et al*, 2014). After fertilization, paternal mitochondria are dissociated from the axoneme and fragmented into small mitochondria, which are then engulfed by autophagosomes. Their degradation partly depends on p62, and accumulation of K63‐linked ubiquitin chains on paternal mitochondria has been observed. Although the precise mechanism remains unclear, this might also involve autophagy regulators during early dissociation or fragmentation steps (Politi *et al*, 2014).

In mouse embryos, ubiquitin and autophagy regulators such as LC3, GABARAP, and p62 are detected on paternal mitochondria (Sutovsky *et al,* 1999; Al Rawi *et al*, 2011). Knockdown of p62 or PINK1 in embryos impairs degradation of paternal mitochondria, supporting autophagy‐dependent degradation of ubiquitylated paternal mitochondria (Rojansky *et al*, 2016). Degradation of paternal mitochondria is also impaired by simultaneous knockdown of Parkin and MUL1, a mitochondria‐localized E3 ubiquitin ligase, suggesting that these E3 ligases may function redundantly (Rojansky *et al*, 2016). The fly and worm Parkin mutants exhibit slight or minor defects in degradation of paternal mitochondria (Politi *et al*, 2014; Sato *et al*, 2018), but possible redundancies with other E3 ligases function cannot be excluded. Similar to *C. elegans*, loss of inner membrane potential has been observed in paternal mitochondria in mouse embryos (Rojansky *et al*, 2016). Notably, Fis1 and TBC1D15 act in degradation of paternal mitochondria (Rojansky *et al*, 2016). These observations suggest a significant overlap between paternal mitochondria degradation and mitophagy of damaged mitochondria in somatic cells. However, in contrast to these studies, Luo *et al* argued that paternal mitochondria are not actively removed and persist in embryos at least until the morula stage (Luo *et al*, 2013). Further studies are needed to resolve when and how paternal mitochondria are removed.

## Physiology and pathophysiology of mitophagy

**Figure 5:**
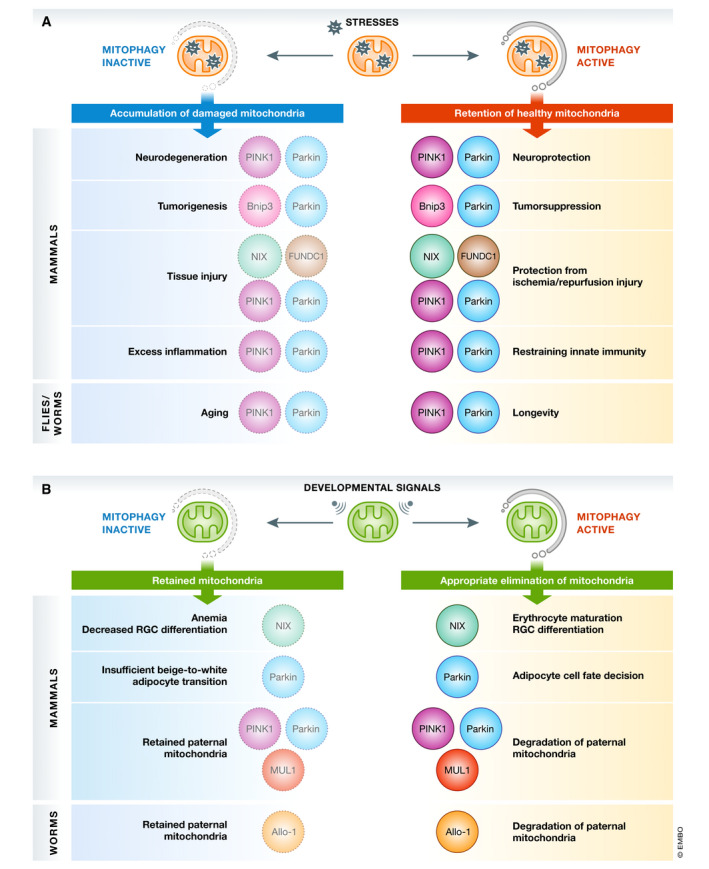
Physiological functions of mitophagy against mitochondrial stresses (A) Mitochondria are constantly challenged by a subset of mitochondrial stresses such as oxidative stress. Mitophagy contributes to mitochondrial quality control and prevention of pathologies including neurodegeneration, tumorigenesis, tissue injury, excess inflammation, and aging. (B) Physiological functions of mitophagy during development and differentiation. Mitochondrial elimination in response to developmental cues is crucial for maturation of cells and tissues.

A growing body of research has explored the pathophysiological functions of mitophagy mainly by using mammalian cells or mice lacking key mitophagy‐related factors. These studies also provide a framework for physiological functions of mitophagy and unveil previously unappreciated links to diverse biological processes (Fig 5).

### Physiological functions of mitophagy in yeast

Since Atg32 is an essential protein for mitophagy in yeast, *atg32*‐null yeast cells have been used to explore the physiological significance of mitophagy. Under longevity‐extending conditions, loss of Atg32 causes accumulation of dysfunctional mitochondria and impaired mitochondrial network, leading to a shortened lifespan (Richard *et al*, 2013). Mitophagy also contributes to the maintenance of mtDNA (Kurihara *et al*, 2011; Karavaeva *et al*, 2017). In heteroplasmic zygotes containing wild‐type and mutant mtDNA molecules, mitophagy is activated and further accelerated by the treatment of mitochondrial uncouplers, suggesting that enhanced mitophagy in zygotes could prevent clonal expansion of mutant mtDNA (Karavaeva *et al*, 2017). Furthermore, during prolonged nitrogen starvation, cells lacking Atg32 exhibit mitochondrial ROS accumulation and mtDNA instability, indicating that mitophagy contributes to mitochondrial fitness under stress conditions (Kurihara *et al*, 2011).

### Mitophagy in development, differentiation, and tissue protection

One example that illustrates the physiological function of mitophagy in mammals is NIX‐mediated mitochondrial elimination during erythrocyte maturation (Schweers *et al*, 2007; Sandoval *et al*, 2008). Intracellular organelles including mitochondria are removed when reticulocytes differentiate into mature erythrocytes. Electron microscopic analysis revealed that during early stage of erythrocyte differentiation, autophagic bodies accumulated in human peripheral blood cells, rat erythroblasts, and reticulocytes (Takano‐Ohmuro *et al*, 2000). Mice lacking the autophagy gene *Atg7* in the hematopoietic system suffer from severe anemia, and *Atg7*‐deficient erythrocytes accumulate damaged or dysfunctional mitochondria with altered membrane potential (Mortensen *et al*, 2010). Consistent with these observations, loss of NIX in mice causes defects in mitochondrial clearance and anemia (Schweers *et al*, 2007; Sandoval *et al*, 2008). NIX is also involved in mouse retinal ganglion cell (RGC) differentiation (Esteban‐Martinez *et al*, 2017). During RGC differentiation, a shift from oxidative phosphorylation to glycolysis is needed in order to meet the metabolic demands of RGCs (Galvan‐Pena & O'Neill, 2014; Ng *et al*, 2015; Chandel *et al*, 2016). Retinas from NIX‐deficient mice show increased mitochondrial mass, reduced expression of glycolytic enzymes, and inefficient neuronal differentiation (Esteban‐Martinez *et al*, 2017).

Mitophagy has also been linked to the maturation of muscle tissue. During myogenesis and muscle regeneration, mitochondrial activity is drastically increased (Duguez *et al*, 2002; Sin *et al*, 2016), likely due to a shift in metabolism from glycolysis to oxidative phosphorylation which eventually increases mitochondrial oxidative stress. Suppression of the essential autophagy gene *Atg5* leads to accumulation of abnormal mitochondria and inefficient differentiation into mature muscle tissue (Sin *et al*, 2016).

Parkin‐dependent degradation of mitochondria has also been linked to cell fate decision of adipocytes. Mice lacking Parkin retain mitochondrial abundance in beige adipocytes and show defects in beige‐to‐white adipocyte transition (Lu *et al*, 2018). While white adipocytes containing a small quantity of mitochondria serve as fat tissues to store energy, beige adipocytes contain a large quantity of mitochondria and act in thermogenesis by uncoupling mitochondrial proton gradient in response to various cues such as chronic cold exposure and exercise (Harms & Seale, 2013; Kajimura *et al*, 2015). After withdrawal of such stimuli, beige adipocytes acquire white adipocyte‐like characteristics in a manner dependent on autophagic mitochondrial turnover (Altshuler‐Keylin *et al*, 2016).

In addition to its roles in development and differentiation, mitophagy is also involved in tissue protection against several types of injuries. Studies in mice demonstrated autophagy‐ and mitophagy‐dependent protection against ischemia/reperfusion (I/R) injury in several tissues. While reperfusion to restore blood flow after ischemia is necessary to salvage the injured tissues, this can paradoxically lead to an excess ROS production from mitochondria (Pulsinelli & Duffy, 1983; Aronowski *et al*, 1997). Genetic suppression of core autophagy‐related genes aggravates neuronal injury and cellular death after I/R injury, mainly by increasing cytochrome c release from mitochondria (Zhang *et al*, 2013). NIX expression in Nix‐deficient neurons restored cell viability after I/R‐induced injury and knockout of NIX in mice exacerbated I/R brain injury as indicated by increased cerebral infarct volume (Yuan *et al*, 2017). FUNDC1 seems to function in cardioprotection through modulating the platelet activity (Zhang *et al*, 2016b). I/R‐induced platelet activation and release of platelet‐derived mediators aggravate tissue injury in the heart (Gawaz, 2004). Hypoxia induces FUNDC1‐mediated mitophagy in platelets, thereby promoting turnover of mitochondria and suppressing platelet activation (Zhang *et al*, 2016b). Parkin plays a protective role in heart against myocardial infarction, as Parkin‐deficient mice exhibit accumulation of dysfunctional mitochondria, a broader zone of the infarction, and reduced survival rates (Kubli *et al*, 2013). PINK1 KO mice also exhibit dysregulated mitochondrial functions and excess cardiomyocyte cell death (Billia *et al*, 2011).

In addition, mitophagy contributes to kidney homeostasis and protection against acute kidney injury (AKI). PINK1‐, Parkin‐, and double‐KO mice show increased mitochondrial damage, ROS generation, inflammatory response, and serum creatinine (an index of renal dysfunction), raising the possibility that PINK1/Parkin‐mediated mitophagy prevents cell death and maintains renal function against I/R‐induced AKI (Tang *et al*, 2018). The autophagy receptor optineurin has also been suggested to act in renal tissue protection (Chen *et al*, 2018). Deletion of optineurin drastically decreases mitophagosome formation during high glucose treatment and exacerbates RTEC (renal tubular epithelial cells) senescence (Chen *et al*, 2018), one of the factors contributing to renal injury in diabetic kidney.

Alcoholic liver disease (ALD) is caused by excess alcohol intake (Rehm *et al*, 2013). Several studies suggest that autophagy‐related processes, especially mitophagy, act in protection against alcohol‐induced liver injury (Ding *et al*, 2010a; Ding *et al*, 2011; Williams & Ding, 2015). Since ALD pathology is associated with ROS accumulation and mtDNA damage, it is conceivable that mitophagy is induced in the liver tissue of ALD patients. Consistent with this idea, Parkin KO mice display increased liver injury, oxidative stress, and steatosis after alcohol treatment, highlighting a protective role of mitophagy against tissue injury in the liver (Williams *et al*, 2015).

Nonalcoholic fatty liver disease (NAFLD) is the most common cause of liver disease and has also been linked with mitochondrial dynamics and Parkin‐independent mitophagy (Loomba & Sanyal, 2013; Masuoka & Chalasani, 2013; Yamada *et al*, 2018). In the NAFLD mice model, megamitochondria, which are extremely enlarged mitochondria, are observed (Wakabayashi, 2002; Neuman *et al*, 2014; Targher *et al*, 2018; Younossi *et al*, 2018). Deletion of Opa1 (an IMM protein required for mitochondrial fusion) in NAFLD model mice decreases mitochondrial size and ameliorates liver tissue damage, suggesting that restoring mitochondrial size can be a potent therapeutic treatment (Yamada *et al*, 2018). In hepatocytes, the autophagy adaptor p62 recruits Keap1, a component of the cullin–RING ubiquitin ligase complex containing the E3 enzyme Rbx1, to mitochondria and promotes ubiquitylation of OMM proteins and mitophagy in a manner independent of Parkin (Yamada *et al*, 2018).

### Mitophagy and cancer

Since accumulation of dysfunctional mitochondria is involved in tumorigenesis, it is conceivable that mitophagy seems to be important as a tumor‐suppressive system (Gogvadze *et al*, 2008; Vara‐Perez *et al*, 2019). Expression of the mitophagy receptor BNIP3 declines in several types of cancer and is associated with cancer metastasis and chemoresistance (Erkan *et al*, 2005; Manka *et al*, 2005; Koop *et al*, 2009). Mice lacking *Bnip3* display more rapid tumor growth than wild‐type mice, which can be caused by excess accumulation of dysfunctional mitochondria and elevated ROS production (Chourasia *et al*, 2015).

Expression of Parkin is also lost in many types of cancer (Bernardini *et al*, 2017). Overexpression of Parkin in breast and glioma cells retarded cellular proliferation (Tay *et al*, 2010). However, as Parkin is involved in proteasomal degradation of cyclins, which is fundamentally important for cell cycle control and tumor growth suppression (Staropoli *et al*, 2003; Veeriah *et al*, 2010; Gong *et al*, 2014), Parkin might act via multiple mechanisms to suppress tumor growth. Similar to Parkin, PINK1 overexpression is suggested to attenuate *in vivo* glioblastoma growth (Agnihotri *et al*, 2016).

Although mitophagy factors described above are shown to be dysregulated in cancer patients, it should be noted that whether they act as a tumor suppressor or promoter depends on cellular subtypes and cancer stages (e.g., BNIP3 is also suggested to support melanoma migration (Maes *et al*, 2014)). Further studies are needed to clarify the precise roles of mitophagy in tumorigenesis.

### Mitophagy and neurodegeneration

Mitophagy may contribute to the prevention of neurodegeneration. As discussed above, PD is a major neurodegenerative disease characterized by loss of dopaminergic neurons in the *substantia nigra* (Lotharius & Brundin, 2002). Mutations in the *Pink1* gene have been associated with PD pathogenesis (Valente *et al*, 2001; Valente *et al*, 2002; Geisler *et al*, 2010b). Dopaminergic neurons expressing Pink1 mutants show enlarged mitochondria and undergo cell death (Park *et al*, 2006). Overexpression of Parkin can eliminate mitochondria containing mtDNA mutations in heteroplasmic cybrid cells, ultimately leading to an increase in mitochondria containing wild‐type mtDNA (Suen *et al*, 2010). Loss of Parkin, which by itself does not cause obvious PD pathogenesis in mice (Palacino *et al*, 2004; Stichel *et al*, 2007), synergistically promotes dopaminergic neuron degeneration in mouse models that also contain mtDNA mutations. These mice accumulate dysfunctional mitochondria, supporting the idea that Parkin acts in mitochondrial quality control and neuroprotection (Pickrell *et al*, 2015; Song *et al*, 2017).

Mitophagy may also play a protective role against Alzheimer’s disease (AD), a progressive neuronal disorder characterized by a severe loss of memories and cognitive functions (Querfurth & LaFerla, 2010). Accumulation of insoluble β‐amyloid plaques and formation of neurofibrillary tangles (aggregates of hyperphosphorylated tau proteins) in brain are the major pathological hallmarks of AD (Small *et al*, 2006; Querfurth & LaFerla, 2010). PINK1 protein levels are decreased, and the number of mitochondria is increased in AD model mouse hippocampal neurons (Manczak *et al*, 2018). Overexpression of Parkin in AD model mice decreases β‐amyloid plaques and amyloid‐induced inflammation in hippocampus and cortex, contributing to amelioration of behavioral abnormalities (Hong *et al*, 2014). Pharmacological or genetic stimulation of mitophagy mitigates β‐amyloid plaque formation and tau hyperphosphorylation, and reverses memory impairment (Sorrentino *et al*, 2017; Fang *et al*, 2019).

Similarly, mitophagy may abate progression of amyotrophic lateral sclerosis (ALS), a disease characterized by degeneration of motor neurons, which leads to muscle weakness and paralysis (Evans & Holzbaur, 2018). Mutations in several autophagy‐related proteins including optineurin, which acts downstream of PINK1/Parkin‐mediated mitophagy, have been linked to ALS (Maruyama *et al*, 2010; Cirulli *et al*, 2015). Loss of optineurin decreases LC3 recruitment to damaged mitochondria and impairs subsequent autophagosome formation. One of the ALS‐associated mutations, E478G, is located in the UBAN domain for ubiquitin binding, disturbs targeting of optineurin to damaged mitochondria, and suppresses mitophagosome formation (Wong & Holzbaur, 2014). TBK1, which phosphorylates optineurin and enhances its binding to ubiquitin chains, is also mutated in ALS patients (Freischmidt *et al*, 2015; Oakes *et al*, 2017; Pozzi *et al*, 2017). A disease‐associated mutation in the TBK1 C‐terminal coiled‐coil domain disrupts its interaction with optineurin and possibly affects mitophagy and ALS pathogenesis (Freischmidt *et al*, 2015). It should be noted that optineurin and TBK1 are also crucial to eliminate cytosolic protein aggregates via autophagy and whether the phenotypes are due to impaired elimination of toxic protein aggregates, or mitophagy, or a combination of both is not currently clear.

### Mitophagy and immune response

Autophagy and mitophagy have been linked to the immune response, suppressing overactivation of the NLRP3 inflammasome and subsequent immune response. Inflammasomes are multisubunit protein complexes consisting of NOD‐like receptor (NLR) that induce downstream immune signaling against microbial infection and intracellular damage (Schroder & Tschopp, 2010). As the NLRP3 inflammasome is activated by mitochondrial ROS and mtDNA (Nakahira *et al*, 2011), autophagy‐dependent clearance of mitochondria suppresses overactivation of NLRP3 inflammasome. Sestrin2, a conserved stress‐inducible metabolic protein, protects cells and tissues against excess activation of the NLRP3 inflammasomes (Kim *et al*, 2016). Macrophages isolated from Sestrin2‐deficient mice show hyperactivation of caspase‐1, leading to enhanced secretion of IL‐1β and IL‐18. Sestrin2 localizes to mitochondria upon lipopolysaccharide stimulation and promotes targeting of p62 to damaged mitochondria, thereby contributing to mitophagy during the immune response and preventing prolonged NLRP3 inflammasome activation (Kim *et al*, 2016).

*PINK1* and *Parkin* KO mice have also been shown to be more sensitive to polymicrobial sepsis‐induced multiple organ failure and death (Kang *et al*, 2016). The enhanced sensitivity of these KO mice to lethal sepsis is alleviated by simultaneous depletion of *Nlrp3* (Kang *et al*, 2016). PINK1/Parkin‐mediated mitophagy is also linked to the STING pathway, a major intracellular signaling pathway of the type I IFN in response to cytosolic DNA (Sliter *et al*, 2018). Disruption of mitochondria triggers inflammatory responses via the NLRP3 inflammasome and also via the cGAS‐STING pathway (Rongvaux *et al*, 2014). After exhaustive exercise, PINK1‐ and Parkin‐deficient mice show increased STING activation (Sliter *et al*, 2018). Importantly, inflammatory responses in these mutant mice are abolished by concurrent depletion of STING, supporting the idea that PINK1 and Parkin may prevent release of mtDNA from dysfunctional mitochondria, thereby inhibiting an excess inflammatory response via the STING pathway (Sliter *et al*, 2018). However, loss of *Sting* does not suppress mitochondrial dysfunctions in *Drosophila Pink1/parkin* mutants, raising the possibility that the Pink1/parkin‐mediated processes are not linked to the STING pathway in flies (Lee *et al*, 2020).

A recent study reveals that hepatocyte‐specific FUNDC1 knockout promotes initiation of hepatocarcinogenesis (HCG), whereas FUNDC1 overexpression in hepatocytes suppresses it, suggesting that FUNDC1 acts in prevention of HCG (Li *et al*, 2019). Loss of FUNDC1 causes accumulation of damaged mitochondria and induces release of mtDNA into the cytosol, leading to aggravated activation of inflammasomes. Thus, dysregulated immune response in FUNDC1‐depleted hepatocytes seems to excessively promote hepatocellular proliferation.

### Mitophagy and aging

Health issues associated with aging are of great concern, especially in aging societies. Autophagy has been suggested to be a convergent mechanism whose activity prevents aging and declines with age (Uddin *et al*, 2012; Hansen *et al*, 2018; Nakamura & Yoshimori, 2018). Similarly, the activity of mitophagy in specific brain region decreases during aging, as indicated by an *in vivo* study using transgenic mice expressing mt‐Keima (Sun *et al*, 2015). On the other hand, a study using *Drosophila* expressing mt‐Keima reveals that mitophagic activity increases in aged flight muscle, raising the possibility that age‐related changes in mitophagy vary in some species and/or tissues (Cornelissen *et al*, 2018). Nevertheless, accumulation of excess mtDNA mutations is one of the underlying factors in mammalian aging (Trifunovic *et al*, 2004; Kujoth *et al*, 2005), implying that mitophagy may function to eliminate mitochondria with mutated mtDNA and prevent aging.

The roles of PINK1 and Parkin in aging have extensively been studied in *Drosophila* (Greene *et al*, 2003; Pesah *et al*, 2004). *Parkin*‐null flies are viable but show significantly reduced longevity compared with wild‐type. *Parkin*‐null flies have degenerated muscle tissues and severe defects in locomotor functions, highlighting Parkin as a critical regulator of tissue homeostasis (Greene *et al*, 2003; Pesah *et al*, 2004). Ubiquitous overexpression of Parkin in flies leads to lifespan extension, likely via modulating intracellular proteostasis and mitochondrial dynamics (Rana *et al*, 2013). Notably, neuron‐specific overexpression of Parkin is sufficient to prolong lifespan (Rana *et al*, 2013), whereas *PINK1* mutant flies display shortened lifespan and myopathology (Clark *et al*, 2006; Park *et al*, 2006; Yang *et al*, 2006) .

In *C. elegans*, disruption of mitophagy contributes to progressive accumulation of damaged mitochondria and decreased cellular functions during aging (Palikaras *et al*, 2015). The *daf‐2* insulin/IGF‐1 receptor mutant is generally used as a model of extended lifespan. Strikingly, mitophagy in the long‐lived *daf‐2* mutant is upregulated, and knockdown of mitophagy regulators shortens lifespans of the *daf‐2* mutant, suggesting that mitophagy is critical for lifespan extension of the *daf‐2* mutant (Palikaras *et al*, 2015). Simultaneous knockdown of DCT‐1 or PINK‐1 and SKN‐1, an ortholog of mammalian nuclear factor NRF2 involved in mitochondrial biogenesis, further shortens lifespan, suggesting that coordination of mitochondrial biogenesis and degradation is critical for longevity in worms (Fig 4C).

## Conclusions and future perspectives

Mitophagy deficiency is emerging as a potential cause of various pathologies, and thus, interventions targeting mitophagy may possess therapeutic potential (Georgakopoulos *et al*, 2017). Pharmacological screens to identify chemical agents to modulate elimination of mitochondria are ongoing, and several synthetic and natural chemical compounds including Urolithin A have been shown to facilitate mitophagy (Ryu *et al*, 2016). Moreover, a recent study established AUTAC, an autophagy‐targeting chimera that contains S‐guanylation‐inspired degradation tag for autophagy and a warhead to provide target specificity (Ito *et al*, 2013; Takahashi *et al*, 2019). When AUTAC is targeted to mitochondria, selective clearance of mitochondria via autophagy is induced in a manner independent of PINK1/Parkin, and in turn, biogenesis of functional mitochondria is increased in cells from Down syndrome patients. Very recently, mito‐SRAI, a new mitophagy probe that can be applied to both live and fixed samples, has been developed as a tool for high‐throughput *in vitro* screen for mitophagy chemical inducers and *in vivo* histological analysis in mouse models of neurodegeneration (Katayama *et al*, 2020). Future attempts to identify small molecules that specifically bind and regulate mitophagic factors will aid therapeutic approaches to human disorders associated with mitochondrial dysfunction.

In yeast, Atg32‐mediated mitophagy seems to be the sole pathway that confers selectivity toward mitochondria versus other cellular constituents, acting as a quantity adaptation to the low‐energy demand in non‐dividing cells. In mammals, multiple mitophagy receptors/adaptors promote mitochondrial degradation in certain specific cell types and under particular conditions, but they may also function redundantly in reducing or completely eliminating mitochondria. Since mammalian cells contain a large number of mitochondria that are heterogeneous (e.g., membrane potential, respiratory activity, and oxidative damage), they may have needed to additionally evolve diverse ubiquitin‐mediated pathways that establish selectivity toward dysfunctional mitochondria versus healthy mitochondria, acting as a quality management system. These mitophagy‐dependent mitochondrial quantity and quality control mechanisms are not mutually exclusive, as the former can help improve mitochondrial fitness in cooperation with mitochondrial biogenesis that provides fresh mitochondria, and the latter can help decrease mitochondrial populations without wasting healthy mitochondria.

Over the last decade, numerous studies have contributed to establish the paradigm that mitophagy serves as a system to modulate mitochondrial fitness and populations in response to changes in intra‐ and extracellular environments. Studies using *in vivo* models have provided new insights into the physiological and pathological implications of mitophagy (see Box 2). Although loss of mitophagy is detrimental to mitochondrial homeostasis, it seems conceivable that aberrantly hyperactivated mitophagy could also be deleterious and may ultimately lead to cell death. Therefore, mitophagy must be tightly regulated by both accelerators and brakes. Several outstanding questions remain to be addressed: What are those pro‐ and anti‐mitophagic factors/mechanisms? How is basal mitochondrial turnover controlled? Are there additional mitophagy receptors that are ubiquitous or limited to specific tissues and cell types? How do cells coordinate mitochondrial biogenesis and degradation? Do other organelles promote and/or suppress mitophagy? Undoubtedly, many exciting discoveries and translational innovations are yet to come.

Box 2 **Tools for monitoring mitophagy *in vivo***

**A. mt‐Keima**

Mitochondrial degradation can be analyzed *in vitro* and *in vivo* by fluorescence microscopy using a mitochondrial matrix‐targeted Keima (mt‐Keima) whose excitation spectrum peaking depends on pH (Sun *et al*, 2017). Upon mitophagy, this red fluorescent protein is delivered to lysosomes and changes its excitation peak from 438 nm at neutral pH to 550 nm at acidic pH, which allows for dual‐excitation ratiometric imaging (Katayama *et al*, 2011). Since mt‐Keima is resistant against lysosomal proteases and stays fluorescent at acidic pH, it has been used to provide a readout of mitophagy in mammalian cells and tissues (Bingol *et al*, 2014; Kageyama *et al*, 2014; Mizumura *et al*, 2014; Hirota *et al*, 2015; Ikeda *et al*, 2015; Shirakabe *et al*, 2016a; Shirakabe *et al*, 2016b; Xu *et al*, 2018). Moreover, a transgenic mouse and fly expressing mt‐Keima has been established to evaluate mitophagy *in vivo* under a variety of experimental conditions, revealing that mitophagy activity varies from tissues to tissues (Sun *et al*, 2015; Cornelissen *et al*, 2018).

**B. MitoTimer**

Mitophagy and mitochondrial biogenesis involve a dynamic turnover of mitochondria. Hence, a time‐sensitive fluorescent protein is a good probe to chase changes in mitochondrial dynamics. Fluorescent timer, or DsRed1‐E5, is a redox‐sensitive variant, and its fluorescence shifts over time from green to red as it becomes mature (Terskikh *et al*, 2000). MitoTimer consisting of the timer fluorescent protein fused to the N‐terminal mitochondrial targeting sequence of COX8A has been established and expressed in different tissues (Ferree *et al*, 2013; Hernandez *et al*, 2013; Laker *et al*, 2014; Trudeau *et al*, 2014; Stotland & Gottlieb, 2016; Laker *et al*, 2017). Rates of mitochondrial turnover and MitoTimer fluorescence transmission depend on the balance between import of newly synthesized components and degradation of old materials. Transgenic mice expressing MitoTimer reveal that this fluorescent molecular clock is a good tool to monitor mitochondrial structure, function, oxidative stress, and mitophagy *in vivo* under physiological and pathophysiological conditions (Wilson *et al*, 2017).

**C. Mito‐QC**

Mito‐QC is a pH‐sensitive tandem mCherry‐GFP‐tagged fluorescent marker fused with the C‐terminal TM domain derived from the OMM‐anchored protein FIS1 (Allen *et al*, 2013; McWilliams & Ganley, 2019). Upon mitophagy, mitochondria containing mito‐QC (both mCherry‐ and GFP‐positive) are delivered to lysosomes where mCherry remains resistant against acidic pH and proteases, but GFP becomes quenched under acidic conditions. Thus, mCherry‐only (GFP‐negative) foci can be observed and quantified as indicators of mitochondrial degradation in lysosomes. Recent studies using transgenic mouse models expressing mito‐QC reveal high levels of mitophagy in the developing heart and adult kidney, and PINK1‐independent basal mitochondrial turnover *in vivo* (McWilliams *et al*, 2016; McWilliams *et al*, 2018).

## Conflict of interest

The authors declare that they have no conflict of interest.
